# mRNA vaccines induce rapid antibody responses in mice

**DOI:** 10.1038/s41541-022-00511-y

**Published:** 2022-08-01

**Authors:** Makda S. Gebre, Susanne Rauch, Nicole Roth, Janina Gergen, Jingyou Yu, Xiaowen Liu, Andrew C. Cole, Stefan O. Mueller, Benjamin Petsch, Dan H. Barouch

**Affiliations:** 1 Center for Virology and Vaccine Research, Beth Israel Deaconess Medical Center, Harvard Medical School, Boston, MA 02215 USA; 2 CureVac AG, Tübingen, Germany; 3 Department of Emergency Medicine, Beth Israel Deaconess Medical Center, Boston, MA 02215 USA; 4 Ragon Institute of MGH, MIT and Harvard, Cambridge, MA USA

**Keywords:** RNA vaccines, SARS-CoV-2

## Abstract

mRNA vaccines can be developed and produced quickly, making them prime candidates for immediate outbreak responses. Furthermore, clinical trials have demonstrated rapid protection following mRNA vaccination. Thus, we sought to investigate how quickly mRNA vaccines elicit antibody responses compared to other vaccine modalities. We first compared the immune kinetics of mRNA and DNA vaccines expressing SARS-CoV-2 spike in mice. We observed rapid induction of antigen-specific binding and neutralizing antibodies by day 5 following mRNA (4 µg/mouse), but not DNA (50 µg/mouse), immunization. Comparing innate responses hours post immunization, the mRNA vaccine induced increased levels of IL-5, IL-6, and MCP-1 cytokines which maybe promoting humoral responses downstream. We then evaluated the immune kinetics of an HIV-1 mRNA vaccine in comparison to DNA, protein, and rhesus adenovirus 52 (RhAd52) vaccines of the same HIV-1 envelope antigen in mice. Again, induction of envelope-specific antibodies was observed by day 5 following mRNA vaccination, whereas antibodies were detected by day 7–14 following DNA, protein, and RhAd52 vaccination. Thus, eliciting rapid humoral immunity may be a unique and advantageous property of mRNA vaccines for controlling infectious disease outbreaks.

## Introduction

In comparison to traditional vaccines, novel mRNA vaccines can be developed and produced for distribution in record time. This makes them suitable for rapidly controlling outbreaks as demonstrated in the current severe acute respiratory syndrome coronavirus 2 (SARS-CoV-2) pandemic^1–4^. Furthermore, clinical trials have now shown the rapid protective efficacy of mRNA vaccines post prime immunizations^5,6^. For example, the Pfizer mRNA vaccine clinical trial has demonstrated clear divergence between placebo and vaccine recipients only 12 days after the first dose was administered^5^.

Here, we sought to investigate how quickly mRNA vaccines induce protective humoral immunity in comparison to other vaccine modalities in mice. Specifically, we immunized C57BL/6 mice intradermally as well as intramuscularly with mRNA or DNA vaccines encoding SARS-CoV-2 full-length pre-fusion stabilized Spike protein^7,8^. The mRNA vaccine induced binding as well as neutralizing antibody titers as early as 5 days post immunization. To examine the effect of innate immune triggers, we evaluated the innate cytokine profiles of the two vaccines hours post immunization. Compared to the DNA vaccine, the mRNA vaccine induced a more robust production of IL-5, IL-6, and MCP-1. To determine whether the rapid immune kinetics would translate to other mRNA vaccines of different diseases and antigens, we evaluated the immune kinetics of an mRNA vaccine expressing HIV-1 envelope along with DNA, protein, and Rhesus Adenovirus 52 (RhAd52) vaccines of the same antigen. We were able to observe the rapid induction of antibodies 5 days post mRNA vaccine immunization. The rapid humoral immune kinetics is an advantageous property of the mRNA vaccines, which further supports their use in mitigating infectious disease outbreaks.

## Results

### Rapid induction of binding antibody titers post SARS-CoV-2 Spike mRNA vaccination in mice

**Fig. 1:**
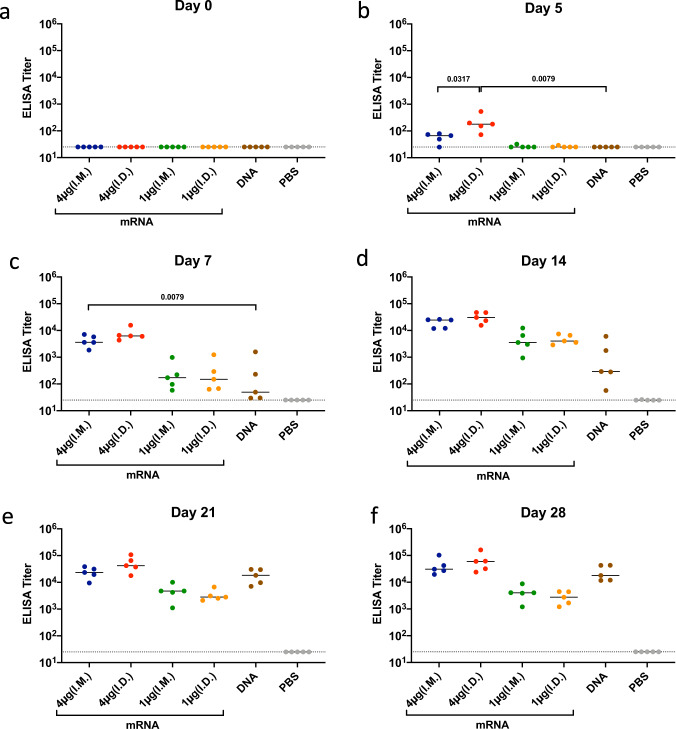
Kinetics of binding antibody responses of mice immunized with SARS-CoV-2 Spike mRNA and DNA vaccines. C57BL/6 mice were immunized I.M. or I.D. with spike encoding mRNA vaccine (1 µg or 4 µg/mouse), DNA vaccine (50 µg/mouse), or PBS. Binding antibody titers were assessed via ELISA at 0 (**a**), 5 (**b**), 7 (**c**), 14 (**d**), 21 (**e**), and 28 (**f**) days post immunization. Each dot represents an individual animal, bars depict the median and the dotted line shows the limit of detection. Statistical analysis was performed using Mann–Whitney test. I.M. intramuscular, I.D. intradermal.

To determine the kinetics of humoral immune response, C57BL/6 mice were vaccinated intramuscularly (I.M.) or intradermally (I.D.) with mRNA vaccines expressing SARS-CoV-2 Spike at doses of 1 µg or 4 µg. Additional groups of mice were immunized I.M. with a previously investigated DNA vaccine^8^ at a dose of 50 µg or with PBS as a sham control. Spike-specific binding antibodies were measured in serum by ELISA. Spike-specific binding antibody titers (median 179; range 72–532) were observed by day 5 following I.D. immunization with the 4 µg dose of the mRNA vaccine (Fig. 1). Antibody titers were also observed 5 days post I.M. immunization, although I.D. immunization resulted in higher titers at this early timepoint (*P* = 0.0317). In contrast, the median titers from both low-dose mRNA immunization and DNA vaccination remain below limit of detection on day 5. By day 7, we still observed significantly higher antibody titers for mice immunized I.M. with the 4 µg dose of mRNA compared to mice immunized I.M. with the DNA vaccine (*P* = 0.0079). On day 14, all mRNA groups elicited dose-dependent antibody responses with no significant difference between I.M. and I.D. vaccination. Lower but detectable titers were also observed in the DNA vaccine group, while the sham group antibody titers remained at baseline. By day 21, antibody titers elicited by the DNA vaccine were comparable to those induced by the 4-µg dose mRNA groups. These results demonstrate that mRNA vaccines induce rapid antibody responses and that these responses are dose- and route-dependent.

### Early Induction of neutralizing antibodies upon SARS-CoV-2 Spike mRNA vaccination

**Fig. 2:**
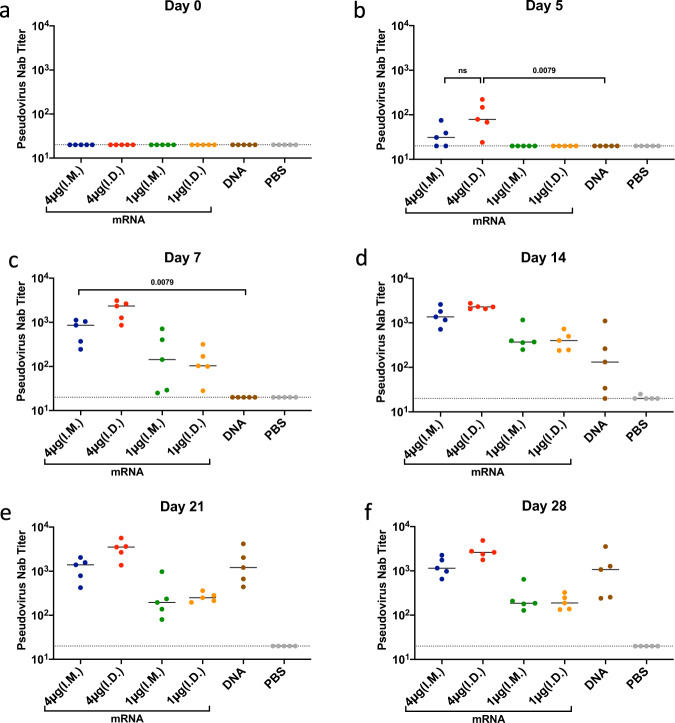
Kinetics of neutralizing antibody titers of mice immunized with SARS-CoV-2 Spike mRNA and DNA vaccines. C57BL/6 mice were immunized I.M. or I.D. with spike encoding mRNA vaccine at 1 µg/mouse or 4 µg/mouse doses, spike encoding DNA at 50 µg/mouse dose or PBS. Neutralizing antibody titers were assessed via pseudovirus neutralization assay at 0 (**a**), 5 (**b**), 7 (**c**), 14 (**d**), 21 (**e**), and 28 (**f**) days post immunization. Each dot represents an individual animal, bars depict the median and the dotted line shows limit of detection. Statistical analysis was performed using Mann–Whitney test. I.M. intramuscular, I.D. intradermal.

Next, we evaluated neutralizing antibody titers via a pseudovirus neutralization assay^9^. Consistent with the binding antibody titer data, mice immunized via either I.M. or I.D. routes at the 4 µg dose of mRNA vaccine exhibited neutralizing antibody (NAb) titers as early as day 5 following immunization (Fig. 2). Mice immunized I.D. with the 4 µg dose exhibited the highest NAb titers on day 5 (median 79; range 24–221). By day 7, mice immunized I.M. with the 4 µg of mRNA vaccine showed significantly higher neutralizing antibody titers than mice immunized I.M. with the DNA vaccine (*P* = 0.0079). The 1 µg dose mRNA vaccine elicited lower NAbs than the 4 µg dose. By day 21, NAb titers induced by the DNA vaccine reached levels that were comparable to those elicited by the 4 µg mRNA vaccine.

### Cytokine and chemokine responses of mRNA and DNA vaccines

**Fig. 3:**
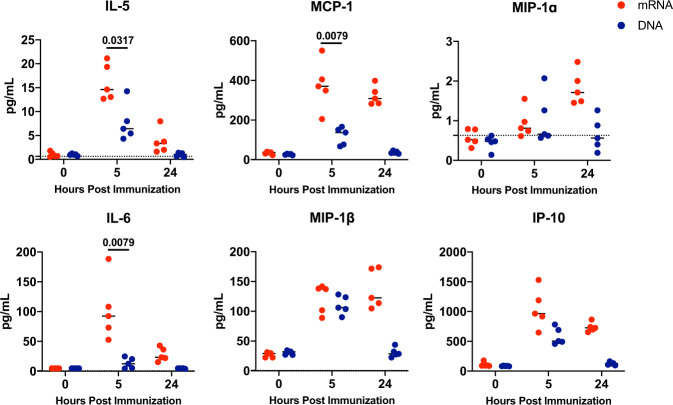
Cytokine and chemokine responses elicited in mice immunized with SARS-CoV-2 Spike mRNA and DNA vaccines. C57BL/6 mice were immunized I.M. with either 4 µg/mouse dose of spike encoding mRNA vaccine or, 50 µg/mouse dose of spike encoding DNA vaccine. Plasma collected at 0, 5 and 24 h post immunization were analyzed for cytokines using a U-PLEX Biomarker Group 1 (ms) 35-Plex kit from Meso Scale Discovery. Each dot represents an individual animal, bars depict the median and the dotted line shows limit of detection. Statistical analysis was performed using Mann–Whitney test. I.M intramuscular.

To better understand differences in innate immune responses post SARS-CoV-2 mRNA or DNA vaccination that may contribute to humoral immune responses, we evaluated the kinetics of expression of cytokines and chemokines at 5 and 24 h post vaccination in mouse plasma samples. We compared mice that were immunized with 4 µg of mRNA vs 50 µg of DNA via I.M. injection routes. Cytokines that play a key role in the initiation and development of B cells and antibody production were significantly induced following mRNA vaccination (Fig. 3). IL-5, which is a critical cytokine for mouse B-cell differentiation to antibody-secreting plasma cells^10^, was higher (*P* = 0.0317) in mRNA vaccinated mice (median 14.62 pg/mL) than in DNA vaccinated mice (median 6.41 pg/mL) at 5 h following immunization (Fig. 3). Similarly, IL-6, which is critical for B-cell proliferation and isotype switching, was higher (*P* = 0.0079) 5 h following mRNA vaccination (median 92.37 pg/mL) compared to DNA vaccination (median 12.43 pg/mL)^11^. Furthermore, MCP-1, MIP-1ɑ, MIP-1β as well as IP-10, which are key chemokines for antigen-presenting cell activation and migration, were also induced at 5 and 24 h after mRNA immunization.

### Humoral and cellular immune kinetics in mice immunized with HIV-1 env mRNA vaccine

**Fig. 4:**
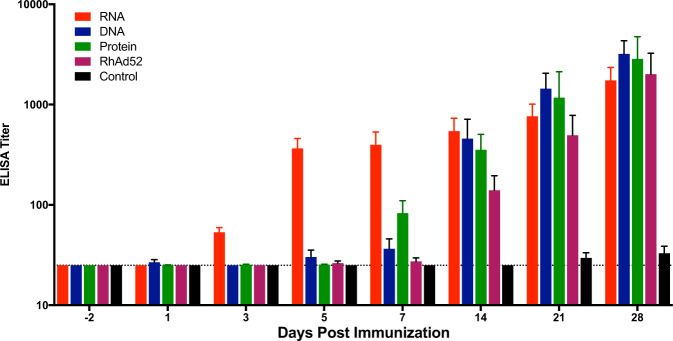
Rapid humoral immunity of mRNA vaccine expressing HIV-1 Env gp140WT compared to DNA, RhAd52 and protein vaccine modalities of the same antigen. C57BL/6 mice were immunized I.M. with mRNA (15 µg), DNA (50 µg), rhesus adenovirus 52 (RhAd52) (10^9^ viral particles) or protein (50 µg +100 µg Adju-phos (InvivoGen)) vaccines that encode or represent the HIV-1 env antigen. Antigen-specific binding antibodies were assessed at −2, 1, 3, 5, 7, 14, 21, and 28 days post immunization via ELISA. Bars depict the median + /− the standard error of the mean (SEM) and the dotted line shows limit of detection. Statistical analysis was performed using Mann–Whitney test. I.M. intramuscular.

We next sought to determine whether the rapid kinetics observed with SARS-CoV-2 Spike mRNA vaccine could be generalizable to mRNA vaccines encoding other antigens. We evaluated the antibody kinetics of an mRNA vaccine (15 µg/mouse) encoding a Clade C 459 C HIV-1 envelope (Env) gp140, as well as a DNA vaccine (50 µg/mouse), purified protein vaccine (50 µg/mouse with 100 µg Adjuphos adjuvant), and rhesus adenovirus 52 (RhAd52) vaccine (10^9^ viral particles/mouse) with the same HIV Env antigen. These vaccines were all delivered I.M. Low antibody titers (median 52; range 32–66) were observed on day 3 following HIV-1 Env mRNA immunization, but robust antibody responses (median 356; range 114–689) were evident on day 5 following HIV-1 mRNA vaccine immunization, whereas DNA, protein, and RhAd52 vaccines were largely baseline at that timepoint (*P* = 0.0079) (Fig. 4). By day 21, all four vaccine modalities showed similar robust antibody titers. These data suggest that immune response kinetics after mRNA vaccination are more rapid than with three other leading vaccine technologies.

**Fig. 5:**
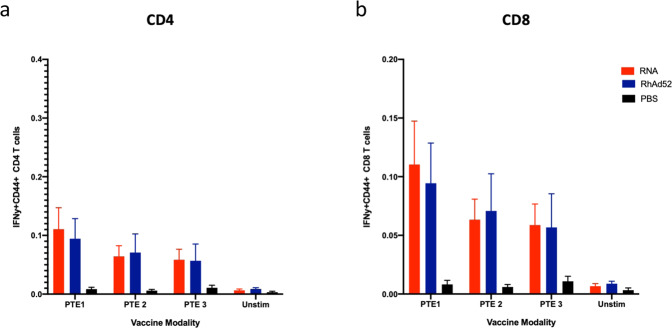
IFNγ-positive CD4 and CD8 T-cell responses 1 week post HIV-1 Env gp140WT mRNA and RhAd52 immunizations in mice. C57BL/6 mice were immunized I.M. with mRNA (15 µg), rhesus adenovirus 52 (RhAd52) (10^9^ viral particles) or PBS. Spleen samples were collected 1 week post immunization and splenocytes were stimulated with HIV-1 Env peptide pools PTE 1–3. IFNγ+ T cells (CD4 (**a**) and CD8 (**b**)) were stained and analyzed by high-parameter flow cytometry. The bars depict the median + /− the standard error of the mean (SEM). I.M. intramuscular.

To examine differences in cellular immune responses between mRNA and RhAd52 vaccine modalities, we measured IFNγ positive CD4 and CD8 T cells 1 week post immunization. At this early timepoint, we did not observe significant differences in responses between the two modalities (Fig. 5).

## Discussion

Novel mRNA vaccines allow for faster development and large-scale production compared to other traditional vaccine methods. Hence, they have demonstrated remarkable utility in mitigating the SARS-CoV-2 pandemic. Furthermore, clinical trials have demonstrated that mRNA vaccines may also provide more immediate protection post prime vaccination^5^. To investigate this further, we examined the humoral immune kinetics of SARS-COV-2 and HIV-1 mRNA vaccines in comparison to other types of vaccines. Various studies have now demonstrated that vaccine-elicited humoral immune responses strongly correlate with protection against SARS-CoV-2 challenge^12,13^. We found that both mRNA vaccines elicited rapid antibody responses by day 5 following immunization. Although antibody responses post SARS-COV-2 mRNA vaccination have previously been reported 1 week post prime immunizations^14^, here we show an even earlier response at 5 days post immunization in this model. Furthermore, in addition to comparing the kinetics of mRNA vaccines head-to-head with other vaccine types, including DNA, protein and RHAd52, we demonstrate that rapid humoral immunity is independent of antigen encoded in the mRNA.

Vaccines have previously been altered to improve the speed and magnitude of IgG responses in mice. For example, a rabies virus vaccine has previously been modified to express a B-cell activating factor (BAFF), to induce IgG antibodies by day 5 that was not observed without the adjuvant^15^. More importantly, improving the antibody kinetics significantly improved the survival of mice challenged with Rabies virus^15^.

Similarly, co-stimulation of an inactivated flu vaccine with TLR-7 agonist (Imiquimod) has previously been shown to accelerate antigen-specific antibody production^16^. IgG antibody secretion in peritoneal B cells was observed in 5 days with the adjuvant, while these antibodies were not induced solely with the inactivated virus. Imiquimod is demonstrated to accelerate the vaccine-elicited antibody production by inducing potent activation and differentiation of B cells into antigen-specific antibody producing cells^16^. This study brings into question whether mRNA vaccines are self-adjuvanted to trigger TLR-7 or other innate sensors that induce rapid humoral immunogenicity.

The early antibody responses observed our experiments were both dose and route-dependent. Intradermal immunization of the mRNA vaccine elicited higher early antibody responses than intramuscular immunization at the 4 µg dose, which is consistent with prior studies that have reported that I.D. immunization of mRNA vaccines results in stronger antibody responses than I.M. immunization^17,18^. This may be due to local antigen-presenting cells (APCs) in the skin, such as dermal dendritic cells, to process and deliver antigen to T and B cells in the draining lymph nodes^19^. In Thailand, I.D. administration of the rabies vaccine for post-exposure prophylaxis (PEP) has offered a cost-effective alternative to I.M. immunizations^20^.

To examine differences in early innate responses induced by mRNA and DNA vaccines, we compared cytokine profiles at 5- and 24-h post immunization. Cytokines that play a key role in B-cell development, such as IL-5 and IL-6, were better induced following mRNA vaccination. IL-5 supports terminal mouse B-cell differentiation to antibody-secreting plasma cells and promotes homeostatic proliferation, survival, and antibody production^10^. IL-6 is critical for B-cell proliferation and isotype switching, which is necessary to produce IgG antibodies that are represented in our antibody titer data^11^. Other cytokines that are upregulated post mRNA vaccine immunization, such as MCP-1, MIP-1ɑ, MIP-1β, as well as IP-10, are important for APC recruitment and activation. MCP-1 recruits, monocytes, memory T cells, and dendritic cells to the sites of inflammation^21^. MIP-1α and MIP-1β are major cytokines produced by macrophages and monocytes during inflammation and promote lymphocyte migration^22,23^. IP-10 is attributed to several roles, such as chemoattraction for monocytes/macrophages, T cells, NK cells, and dendritic cells, promotion of T-cell adhesion to endothelial cells^24^. Overall, the cytokine data suggest that mRNA vaccination induces cytokines that are key in APC recruitment and activation as well as B-cell differentiation and proliferation.

We show that rapid antibody responses were induced not only by mRNA vaccines for SARS-CoV-2 spike but also for HIV-1 envelope, suggesting the generalizability of our findings. mRNA vaccines for HIV-1 have previously been investigated, although the kinetics of early antibody production has not been examined^25^. Our results suggest that rapid humoral response is a characteristic of the mRNA vaccine platform rather than an antigen-specific finding. Other vaccine platforms, such as DNA, RhAd52, and protein vaccines, are also immunogenic but do not show the rapid induction of antibody responses by day 5 following immunization.

In summary, our data show that two different mRNA vaccines induced rapid antibody responses by day 5 following immunization in mice. Future studies will need to determine the generalizability of these observations. Nevertheless, these findings may help explain the rapid protection achieved with mRNA vaccines in clinical trials.

## Methods

### Mice and study designs

Seven- to 8-week-old female C57BL/6 mice (*n* = 5) were purchased from The Jackson Laboratory (Bar Harbor, ME). For SARS-CoV-2 vaccine-based experiments, previously published mRNA (CV2CoV) and DNA vaccines encoding a full-length ancestral SARS-CoV-2 S protein with di-proline mutations were used^8,26–29^. The mRNA vaccine is obtained from CureVac AG, while the DNA vaccine is produced by the Barouch lab^8,29^. The mRNA vaccine was administered (intramuscularly (I.M.) or intradermally (I.D.)) at 1 µg/mouse or 4 µg/mouse doses. The DNA vaccine, expressing the same spike antigen, was I.M. injected at 50 µg/mouse dose. For the HIV-1 vaccine kinetics experiments, groups of mice were immunized with mRNA (15 µg), DNA (50 µg), Rhesus Adenovirus 52 (RhAd52) (10^9^ viral particles) or protein vaccines (50 µg +100 µg Adju phos (InvivoGen)). The HIV-1 mRNA vaccine was also obtained from CureVac AG, while the rest of the vaccines were produced in the Barouch lab. All vaccines encode or represent the same HIV-1 clade C 459 C gp140 env antigen. I.D. administrations were administered at 25 µL dose at two sites while I.M. injections were administered at 50 µL in each of the quadriceps. Blood samples were collected from mice via submandibular bleeds.

### Ethical statement

All animal experiments were conducted in accordance with all relevant local, state, and federal regulations. All studies were approved by the Bioqual Institutional Animal Care and Use Committee (IACUC).

### ELISA

SARS-CoV-2 Spike, as well as HIV-1 Env-specific binding antibodies, were assessed by Enzyme-linked immunosorbent assays (ELISAs)^30,31^. Ninety-six-well plates were coated with 1 µg/ml of SARS-CoV-2 S protein (Sino Biological) or HIV-1 clade C Env 459 C gp140 in 1× Dulbeccoʼs phosphate-buffered saline (DPBS) and incubated at 4 °C overnight. The next day, plates were washed once with wash buffer (0.05% Tween-20 in 1× DPBS) and blocked with 350 µl of casein block per well for 2–3 h at room temperature. Next, the block solution was discarded, and plates were blotted dry. Three-fold serial dilutions of serum in casein block were added to wells, and plates were incubated for 1 h at room temperature. Plates were washed three times and then subsequently incubated for 1 h with 0.1 µg/ml of AffiniPure Rabbit Anti-Mouse IgG or HRP antibody (Jackson Immuno #315-035-045) in casein block at room temperature in the dark. Plates were washed three times, and then 100 µl of SeraCare KPL TMB SureBlue Start solution was added to each well; plate development was halted by the addition of 100 µl of SeraCare KPL TMB Stop solution per well. The absorbance at 450 nm was recorded using a VersaMax or Omega microplate reader. ELISA endpoint titers were defined as the highest reciprocal serum dilution that yielded an absorbance twofold above the background.

### SARS-CoV-2 pseudovirus neutralization assay

The SARS-CoV-2 pseudoviruses expressing a luciferase reporter gene were first generated^8^. Briefly, the packaging construct psPAX2 (AIDS Resource and Reagent Program), luciferase reporter plasmid pLenti-CMV Puro-Luc (Addgene), and S protein expressing pcDNA3.1-SARS-CoV-2 SΔCT were co-transfected into HEK293T cells by lipofectamine 2000 (Thermo Fisher Scientific). The supernatants containing the pseudotype viruses were collected 48 h after transfection; pseudotype viruses were purified by filtration with a 0.45-µm filter. To determine the neutralization activity of the antisera from vaccinated animals, HEK293T-hACE2 cells were seeded in 96-well tissue culture plates at a density of 1.75 × 10^4^ cells per well overnight. Three-fold serial dilutions of heat-inactivated serum samples were prepared and mixed with 50 µl of pseudovirus. The mixture was incubated at 37 °C for 1 h before adding to HEK293T-hACE2 cells. Forty-eight hours after infection, cells were lysed in Steady-Glo Luciferase Assay (Promega) according to the manufacturer’s instructions. SARS-CoV-2 neutralization titers were defined as the sample dilution at which a 50% reduction in relative light units was observed relative to the average of the virus control wells.

### Cytokine analysis

The levels of 35 cytokines in plasma were determined using U-PLEX Biomarker Group 1 (ms) 35-Plex kit from Meso Scale Discovery (MSD, Rockville, MD). Plasma IFN-α and IFN-β levels were tested using individual U-PLEX Mouse IFN-α Assay and U-PLEX Mouse IFN-β Assay kits from MSD. The lower limit of quantification (LLOQ) of 35 biomarkers are listed as follows: EPO (4.5 pg/mL), GM-CSF (0.16 pg/mL), IFN-γ (0.16 pg/mL), IL-1β (3.1 pg/mL), IL-2 (1.1 pg/mL), IL-4 (0.56 pg/mL), IL-5 (0.63 pg/mL), IL-6 (4.8 pg/mL), IL-9 (1.4 pg/mL), IL-10 (3.8 pg/mL), IL-12/IL-23p40 (1.4 pg/mL), IL-12p70 (48 pg/mL), IL-13 (2.7 pg/mL), IL-15 (24 pg/mL), IL-16 (3.6 pg/mL), IL-17A (0.30 pg/mL), IL-17A/F (0.61 pg/mL), IL-17C (2.3 pg/mL), IL-17E/IL-25 (1.6 pg/mL), IL-17F (1.6 pg/mL), IL-21 (6.5 pg/mL), IL-22 (1.2 pg/mL), IL-23 (4.9 pg/mL), IL-27p28/IL-30 (8.7 pg/mL), IL-31 (45 pg/mL), IL-33 (2.2 pg/mL), IP-10 (0.51 pg/mL), KC/GRO (4.8 pg/mL) MCP-1 (1.4 pg/mL), MIP-1α (0.21 pg/mL), MIP-1β (13 pg/mL), MIP-2 β (0.30 pg/mL), MIP-3α (0.10 pg/mL), TNF-α (1.3 pg/mL), VEGF-A (0.77 pg/mL), IFN-α (140 pg/mL), and for IFN-β (5.2 pg/mL). All samples were run in duplicates. Assays were conducted by Metabolism and Mitochondrial Research Core (Beth Israel Deaconess Medical Center, Boston, MA) following the manufacture’s instruction. The assay plates were read by MESO QUICKPLEX SQ 120 instrument and data were analyzed by DISCOVERY WORKBENCH^®^ 4.0 software.

### T-cell immunophenotyping—flow cytometry and intracellular cytokine staining

Lymphocytes were isolated from the spleen were then stained and analyzed by flow cytometry^32^. For ICS staining, cells were re-stimulated in vitro for 1 h at 37 °C with 1 μg/ml of an overlapping HIV-1 Env PTE 1–3 peptide pool. After this incubation, Brefeldin-A and Monensin (BioLegend) were added and samples incubated for an additional 6 h at 37 °C. Cells were subsequently washed, stained, and permeabilized with Cytofix/Cytoperm (BD Biosciences) or Fixation/Permeabilization kit (eBioscience). Surface and cytokine antibodies included: anti-CD4 (FITC, GK1.5, dilution 1/200, Catalogue# 100406), CD44 (BV786, lm7, dilution 1/200, Catalogue# 563736); CD8 (BUV395, 53-6.7, dilution 1/200, Catalogue# 563786) and IFNγ (BV421, XM61.2, dilution 1/200, Catalogue# 505829). All antibodies were purchased from either BioLegend or BD Biosciences. Cells were acquired using an LSR II flow cytometer (BD Biosciences), and data were analyzed using FlowJo (version 9.6.4) software (TreeStar).

### Statistical analyses

Statistical analyses were performed using GraphPad Prism (version 9.0) software (GraphPad Software) and comparison between groups was performed using a two-tailed nonparametric Mann–Whitney *U* *t* test.

### Reporting summary

Further information on research design is available in the Nature Research Reporting Summary linked to this article.

## Supplementary information

REPORTING SUMMARY

## Data Availability

The authors can confirm that all relevant data are included in the paper.
